# Association between Reallocation Behaviors and Subjective Health and Stress in South Korean Adults: An Isotemporal Substitution Model

**DOI:** 10.3390/ijerph17072488

**Published:** 2020-04-05

**Authors:** Saengryeol Park, So-Youn Park, Gapjin Oh, Eun Jung Yoon, In-Hwan Oh

**Affiliations:** 1 Department of Preventive Medicine, School of Medicine, Kyung Hee University, 26, Kyungheedae-ro, Dongdaemun-gu, Seoul 02453, Korea; saengryeol.park@gmail.com; 2 Department of Medical Education and Humanities, School of Medicine, Kyung Hee University, 26, Kyungheedae-ro, Dongdaemun-gu, Seoul 02453, Korea; ukii77@gmail.com; 3 Department of Sport Marketing, Kyung Dong University, 27, Kyundong University-ro, Yanju, Gyeonggido 11458, Korea; jim@kduniv.ac.kr; 4 Department of Physical Education, Korea National University of Education, 250, Cheongju, Chungbuk 27173, Korea; 315836@naver.com

**Keywords:** physical activity, sedentary behavior, adults, mental health, composition analysis

## Abstract

This study used an isotemporal substitution (IS) model to determine the potential reallocation effects of sedentary behavior (SB) and physical activity (PA) on subjective health and stress in South Koreans with data from the Sixth Korean National Health and Nutrition Examination Survey 2015. The analysis included 791 participants whose accelerometer-measured PA was available, divided into three age groups (young adults = 151; mid-age adults = 334; older adults = 306). We adopted SB, light PA (LPA), and moderate-to-vigorous PA (MVPA) to determine how time was allocated to each activity level, then examined the effects of reallocation on subjective health and stress across age groups. The analyses were performed in three steps: single-activity, partition, and IS model. An additional ANCOVA was conducted on statistically significant outcomes (i.e., subjective health of young and older adults). We found that among young adults, reallocating 30 min/week of SB to LPA and to MVPA was linked to high levels of subjective health. In older adults, reallocating 30 min/week of SB and LPA to MVPA was associated with high subjective health. However, this relationship was not observed in mid-age adults. None of the age groups showed a relationship between any activity reallocation and stress. Our findings provide the first insight on the development of interventions aimed at promoting active, healthier lifestyles on the basis of behavior reallocation in South Koreans.

## 1. Introduction

In contemporary society, humans tend to desire and prioritize a healthy life. The World Bank reveals that 10.02% of GDP was invested in health expenditure in 2016 [1]. In South Korea, expenditure has increased continuously from 2004 (4.64%) to 2016 (7.34%). Therefore, public health organizations have made efforts to relieve the social burden of medical costs [2]. However, such efforts often neglect subjective health and stress, both known to drive healthcare usage [3,4,5]. Alarmingly, subjective health is decreasing while stress is increasing. For example, the response rate of good subjective health in South Korea has dropped from 43.95% in 2009 to 32.15% in 2015, however stress rates have increased from 23.80% in 2013 to 29.60% in 2015 [6]. Despite these statistics, we still know little about factors that might influence subjective health and stress in South Korean populations.

Physical activity (PA) plays an important role in our health [7]. For example, objectively measured moderate-to-vigorous PA (MVPA; e.g., running) was higher in those reporting very good health than those reporting fair-to-poor health, but light PA (LPA) had no relation with subjective health [8]. Furthermore, participating in self-reported MVPA and LPA were associated with less stress in adults [9]. In contrast, increased sedentary behavior (SB) is associated with elevated likelihood of all-cause mortality [10] and stress [11], although the association is not always consistent [8].

This connection between PA and health has led to a range of research aimed at improving PA participation and reducing SB, especially because the modern lifestyle is highly sedentary [12]. For example, from 2014 to 2016, MVPA rate decreased in South Korean men (62–53%) and women (55–46%) [6], indicating that individuals spend the majority of their time in SB [11], despite encouragements to meet a set MVPA guideline (150 min/week). Thus, public recommendations of increasing MVPA do not seem to be effective, and another approach might be necessary. A recent PA guideline from the United States suggested a focus on lessening sedentarism through moving more [13], a shift of perspective that emphasizes reallocation of time spent in existing daily activities rather than simply increasing one activity.

The isotemporal substitution (IS) model has emerged as a new method to determine the effects of changing time allocation across activities on a given outcome variable [14], while adjusting for covariates [15]. Data from the IS model helps health professionals develop PA-promoting strategies within a finite and fixed amount of time [16]. For example, reallocating time from SB or light PA (LPA) to MVPA was positively associated with quality of life in older adults [17] and middle-aged Japanese adults [18]. In children, substituting 30 min/day of SB with MVPA improved body composition [19]. Data based on self-reports indicated that reallocating 60 min/day of work to PA was favorably associated with less stress in older adults [20]. A study using self-reported PA also found a similar relationship [20], but the inherent unreliability of self-reports may influence the conclusions drawn on stress using IS models [21]. Currently, little IS-based data are available, and available research on subjective health [15] and stress [22] is not necessarily generalizable to South Koreans. We also know little about age-related variance in PA participation [15]. In summary, a specific, IS-based approach may be needed for developing practical suggestions that encourage individuals to shift their time allotment in various activities that relate to subjective health and stress.

Thus, the aim of this study was to investigate how subjective health and stress are affected when time spent in SB is reallocated to LPA or MVPA. We also examined the effect of increasing MVPA at the expense of LPA. This study is the first attempt at using the IS model on accelerometer-measured PA from a national dataset of South Koreans, instead of relying on self-reports. This study is also the first to include age-related variance in the analysis. Unlike previous studies that reallocated PA time within a single day (e.g., 30 min/day) [19], we reallocated weekly PA because such a schedule change should result in easier adherence. To better understand the complex influence of PA on subjective health measures, we also grouped participants by whether they were more sedentary or active and assessed the effects of PA reallocation within these subgroups.

## 2. Materials and Methods

### 2.1. Data Collection and Participants

This study used data from the Sixth Korean National Health and Nutrition Examination Survey (KNHANES Ⅵ 2015), collected by the Korea Center for Disease Control. The questionnaire, comprising a health interview, health examination, and nutrition survey, was administered by trained medical staff either at the mobile examination center or at participants’ homes [23,24]. KNHANES collects annual data on ~10,000 nationally representative, non-institutionalized individuals aged 1 year old and over [24]. Individuals who resided in South Korea were selected using a multi-stage clustered probability design, in which 192 out of 200,000 primary sampling units in South Korea were chosen, and 20 final target households were selected in each primary sampling unit. Sampling procedures accounted for the inverse of selection probabilities and response rates, and then stratified data by gender and age. Participant who could not engage in PA due to physical limitations were exempt from wearing accelerometers. The original number of participants for this study was 7380; participants who volunteered to wear the accelerometer were included in the final analysis (N = 791). Socioeconomic status, mental health, and anthropometric measures were included. Volunteers were recruited to measure PA and SB at the mobile examination center. For home measurements, participants who agreed to wear accelerometers were given detailed explanations on the study’s purpose and provided written consent [25]. Data from participants who did not answer the survey were coded as missing. Participants wore an accelerometer on either their left or right hip for 7 consecutive days, except when sleeping and participating in any water-related activities.

This research was approved by the University’s Institutional Review Board (IRB no. KHSIRB-19-214 (EA)).

### 2.2. Variables

#### 2.2.1. Subjective Health 

This was measured with one item used in a previous study of South Koreans [26]: “In general, how would you rate your health?” Participants answered with a five-point Likert scale (1 = very good, 2 = good, 3 = moderate, 4 = bad, 5 = very bad) [22]. The scale was reversed (1 = very bad, 2 = bad, 3 = moderate, 4 = good, 5 = very good) to address that high scores show high levels of subjective health. 

#### 2.2.2. Stress 

This was assessed using one item used in a previous study of South Koreans (“What is your general stress level?”) [26], and participants answered using a four-point Likert scale (1 = very high, 2 = high, 3 = low, 4 = very little) [24]. The scale was reversed during analysis (1 = very little, 2 = low, 3 = high, 4 = very high) to be more intuitive (high scores = high stress). 

#### 2.2.3. Physical Activity and Sedentary Behavior 

Accelerometers (model: wGT3X+; ActiGraph, Pensacola, FL, USA) were programmed to start recording PA and SB from the day after participants visited the mobile center, continuing for 7 consecutive days. One individual had only 5 days of data but was not excluded because the amount is within the range of valid days, including weekends [27]. The mean wear time was 5214 min/week. Recordings of at least 10 h/day (± 2 min) were considered valid for analysis. Activity data were classified into MVPA (≥2020 counts per minute (cpm)), LPA (100−2019 cpm), and SB (<100 cpm) [28]. These cutoff scores have been widely applied to adult populations [29].

#### 2.2.4. Covariates 

Statistical models adjusted for age, waist circumference (cm), gender, and wear time of accelerometers. Waist circumference to the nearest 0.1 cm was assessed at the midpoint between the lower border of the rib cage and the iliac crest.

#### 2.2.5. Demographics

Participants recorded their income status and education levels. Income levels were estimated on a four-point Likert scale (low, mid/low, mid/high, high) and categorized into quartiles. Education levels were categorized into primary school, middle school, high school, and university or over.

### 2.3. Statistical Analysis

Ordinal logistic regressions were performed to test the effects of SB, LPA, and MVPA on subjective health and stress, using single-activity, partition, and IS models [8]. All models were run by age groups (young adults 19–29, mid-age adults 30–49, older adults 50–64). The single-activity model separately tested SB, LPA, and MVPA while adjusting for gender, age, waist circumference, and wear time (how long an individual used the accelerometers) to assess the effect of activity on outcome variables. The partition model simultaneously examined SB, LPA, and MVPA while adjusting for gender, age, and waist circumference, but not wear time. The IS model replaced a target behavior while holding other activities constant and adjusting for covariates [12]. For example, when SB was the target behavior, it was omitted from the IS model and 30 min/week was added to LPA, while MVPA and wear time remained constant. To verify described positive effects of higher PA on health outcomes [23], the analysis replaced SB with LPA and MVAP, as well as LPA with MVPA. 

In addition to the main analysis, participants were classified into “High” and “Low” groups that showed statistically significant outcomes in the ordinal logistic regressions based on a previous study [30]. This categorization used average time (min/week) spent on SB (2584 young adults; 3047 mid-age adults; 3007 older adults), LPA (864 young adults; 1344 mid-age adults; 1409 older adults), and MVPA (185 young adults; 181 mid-age adults; 200 older adults). Participants were subsequently clustered into three sets of four subgroups: (1) SB high–LPA low, (2) SB high–LPA high, (3) SB low–LPA low, (4) SB low–LPA high, (5) SB high–MVPA low, (6) SB high–MVPA high, (7) SB low–MVPA low, (8) SB low–MVPA high, (9) LPA high–MVPA low, (10) LPA high–MVPA high, (11) LPA low–MVPA low, (12) LPA low–MVPA high. One-way between-group analysis of covariance (ANCOVA) with a post hoc Bonferroni test was performed to compare subgroup means to examine generality of current study using the mean of activities [30,31]. As with the IS model, all associations were adjusted for gender, age, waist circumference, and wear time.

The required sample size was calculated using a formula suggested by Tabachnick and Fidell [31]: *N* > 50 + 8 × *m* (*m* is the number of independent variables). Accordingly, 98 participants were minimally required to run analyses on each group. Considering correlations with other variables, the current participants of each group (range *N* = 151–334) were thought to be acceptable to perform analyses. Significance was set at P < 0.05. All analyses were conducted in SAS version 9.4 (Statistical Analysis System, SAS.com, Cary, NC, USA).

## 3. Results

The final analysis included 791 participants from three age groups (young adults = 151, mid-age adults = 334, older adults = 306) (Table 1). The mid/low category was the most common income level, and while low income was the least common. Most participants had high school or university education. Waist circumference increased with age. Young adults were the most sedentary, while older adults scored highest in LPA and MVPA. The three activity variables (SB, LPA, MVAP) were lowly to moderately correlated (Pearson’s R = 0.12–0.43). 

Among young adults (Table 2), SB was negatively and MVPA positively associated with subjective health in the single-activity model. In the partition model, MVPA was positively associated with subjective health. In the IS model, reallocating 30 min/week of SB to LPA and to MVPA were both positively associated with subjective health. For older adults, MVPA was positively associated with subjective health in both the single-activity and partition models. In the IS model, reallocating 30 min/week of SB to MVPA and LPA to MVPA were positively associated with subjective health. We did not find any associations among mid-age adults. Subjective health of young and older adults did not significantly differ across activity levels under ANCOVA.

Stress was not associated with any activity in any age group under the three models (Table 3). 

## 4. Discussion

In this study, we demonstrated that spending more time on higher-intensity PA influenced subjective health in a South Korean population. We also showed that IS models are effective in assessing PA effects across different age groups. Our findings should be useful for developing PA-promotion strategies targeted toward different sub-populations. Further interventions could test IS-specific PA guidelines for improving subjective health, including reallocating SB to LPA and MVPA among young adults, as well as shifting more SB and LPA to MVPA among the elderly. 

Reallocating 30 min/week from SB to LPA and to MVPA had small, beneficial effects on subjective health in young adults, but this outcome was absent among mid-age adults, even when more time was reallocated to LPA and MVPA. In contrast, increasing MVPA at the expense of SB was favorably associated with health-related quality of life in Australian mid-age adults, but increasing LPA at the expense of SB resulted in a negative association [17]. Reallocating effects could vary depending on outcome variables or population type [16], due to the discrepancy of PA participation across age groups [28]. Our results here provide the first evidence of age-related variation in reallocation effects and subjective health. Specifically, young adults may be particularly sensitive to even minor time reallocations in LPA, compared with older adults. Based on our findings, we recommend testing age-specific interventions to improve subjective health. For middle-aged working adults, such interventions include standing desks or walking in place for 10 min to encourage LPA. For young adults where MVPA should be encouraged, intervention could include promoting easy sports (e.g., badminton or running) rather than SB during the weekend.

Reallocating 30 min/week from SB to MVPA and from LPA to MVPA had beneficial effects on subjective health in older adults, in line with previous research showing that spending 30 min/day on LPA instead of SB improved walking ability in older adults [32]. Our results increased current understanding on the role of reallocating PA in elderly South Koreans. Because MVPA participation rate is low among older adults [27], healthcare professionals tend to recommend LPA [33]. However, our study suggests that MVPA is more effective than LPA at increasing subjective health. Encouraging older adults to participate more in MVPA may require tailored, IS-based interventions, while replacing SB and LPA with MVPA. A previous intervention aimed at reducing time spent in SB did not directly increase time spent on MVPA [34], indicating the independence of these two activity types. One behavioral change to suggest among older adults is walking downstairs, which is effective in improving physical strength and easy to implement [35]. 

We did not expect that stress in all age groups would be unaffected by reallocating 30 min/week from SB to PA, as this directly contradicts previous findings. Reallocating 30 min/day of accelerometer-measured SB to LPA and MVPA in Japanese adults [36] and reallocating 60 min/day of work time to self-reported PA in Australian older adults [20] were both associated with less stress. Our use of a very small time displacement (30 min/week) may explain this surprising difference between studies, implying that future studies should implement a bigger shift in PA time-use to see age-specific differences on stress.

A limitation of this study is the small magnitude of observed associations. Nevertheless, our results were notable in providing the first direct evidence that even slightly increasing PA can improve subjective health. In addition, although the study used one-item scales for outcome variables, they have all been widely validated [24]. Another limitation is that subjective health may not fully reflect actual participant health, although we note its prevalent usage in research on South Korean populations [37]. We recommend that future studies adopt objective health measures such as physical function. Because this study is cross-sectional, we could not draw any causational conclusions, a limitation that can be addressed through future intervention studies. Moreover, although variation in sleep habits could account for lifestyle differences, we did not include sleep time due to limitations from the original source, where the IS model was tested using only activity variables (SB, LPA, and MVPA) [18]. In addition, despite their accuracy in measuring PA and SB duration, accelerometers are limited when it comes to assessing types of PA and SB [38]. Thus, we recommend the simultaneous use of accelerometers and self-reports to better understand PA and SB. Finally, we did not find significant associations with stress, an unexpected outcome that warrants further investigation to verify whether different time reallocations (e.g., 60, 120 minute/week) would alter the outcome. Despite these limitations, our study is an impetus for more research that applies IS models on national health data in South Korea. 

## 5. Conclusions

In summary, this study was the first to apply IS models on subjective health and stress in the South Korean population. Reallocation of 30 min/week from SB to LPA and MVPA improved subjective health among young adults, while reallocating the same amount of time from SB and LPA to MVPA improved subjective health among older adults. These results provide novel insight on PA promotion and benefit efforts to manage subjective health for both age groups. Specifically, our findings indicate that time reallocation is a valid and flexible method that can be fine-tuned based on subpopulation characteristics. Future studies could translate these patterns into practical guidelines through a fine-tuned investigation of specific time periods and strategies using the IS model, allowing for age-specific increases in PA and reductions in SB.

## Figures and Tables

**Table 1:** Characteristics of participants.

Variable		Age Range			Total
		Young Adults (19−29)	Mid-Age Adults (30−49)	Older Adults (50−64)	
Gender (N)	Male	67	107	108	282
	Female	84	227	198	509
Subtotal		151	334	306	791
Income	Q1 low	37	79	70	186
	Q2 mid/low	42	90	85	217
	Q3 mid/high	37	81	74	192
	Q4 high	33	84	77	194
Subtotal		149	334	306	789
Education	1 below primary school	1	2	47	50
	2 middle school	1	16	62	79
	3 high school	89	127	105	321
	4 university or over	59	186	90	335
Subtotal		150	331	304	785
Waist	N	151	334	305	790
	Mean	77.19	80.66	82.75	80.81
	SD	9.89	10.05	8.97	9.81
Sedentary behavior minutes per week (minutes/day)	N	151	334	306	791
	Mean	2583.48 (369)	3047.37 (435)	3006.94 (430)	
	SD	1103.70	983.93	972.03	
Light physical activity minutes per week (minutes/day)	N	151	334	306	791
	Mean	863.48 (123)	1343.99 (192)	1409.41 (201)	
	SD	490.79	472.04	541.67	
Moderate-to-vigorous activity minutes per week (minutes/day)	N	151	334	306	791
	Mean	185.03 (26)	180.57 (26)	200.25 (29)	
	SD	152.81	150.81	154.50	
Accelerometer wear time minutes per week (minutes/day)	N	151	334	306	791
	Mean	6143.17 (878)	5049.20 (721)	4935.34 (705)	
	SD	1639.40	1324.08	1424.86	

**Table 2:** Single-activity, partition, and isotemporal (IS) models examining associations of sedentary behavior (SB), light physical activity (LPA), and moderate-to-vigorous physical activity (MVPA) with subjective health.

Outcomes	Model	Target Behavior Replaced	Odds Ratio (95% CI)	Odds Ratio (95% CI)	Odds Ratio (95% CI)
			SB	LPA	MVPA
Subjective health (young adults 19–29)	Single-activity	-	0.99921 (0.99994−0.99985) *	1.00102 (1.00216−0.99987)	1.00237 (1.00465−1.00008) *
	Partition	-	0.99965 (1.00002−0.99929)	1.00073 (1.00155−0.9999)	1.00227 (1.00445−1.00009) *
	IS (+ 30)	SB with LPA	-	1.00121 (1.00237−0.00004) *	1.00274 (0.00506−0.00041) *
		SB with MVPA	-	1.00121 (1.00237−1.00004) *	1.00274 (1.00506−1.00041) *
		LPA with MVPA	0.99937 (1.00012−0.99861)	-	1.00186 (1.00423−0.99949)
Subjective health (mid-age adults 30–49)	Single-activity	-	0.99993 (0.00033−0.99953)	0.99986 (1.0005−0.99922)	0.00012 (.00154−0.99869)
	Partition	-	1.00011 (0.00033−0.99989)	1.00009 (1.00057−0.99961)	1.00034 (1.00173−0.99894)
	IS (+ 30)	SB with LPA	-	0.99986 (1.0005−0.99922)	1.00011 (.00153−0.99869)
		SB with MVPA	-	0.99986 (1.0005−0.99922)	1.00011 (1.00153−0.99869)
		LPA with MVPA	0.99993 (1.00038−0.99949)	-	1.00003 (1.0016−0.99845)
Subjective health (older adults 50–64)	Single-activity	-	0.99971 (1.0001−0.99931)	1.00009 (1.00074−0.99943)	1.00181 (1.0039−1.00033) *
	Partition	-	0.9994 (1.00018−0.9997	1.00018 (1.00061−0.99975)	1.00181 (1.00327−1.00041) *
	IS (+ 30)	SB with LPA	-	1.00025 (1.00091−0.99959)	1.00191 (1.00341−1.00041) *
		SB with MVPA	-	1.00025 (1.00091−0.99959)	1.00191 (1.00341−1.00041) *
		LPA with MVPA	0.99982 (1.00023−0.99941)	-	1.00165 (1.00317−1.00012) *

Note. * *p* < 0.05, CI = confidence interval, IS = isotemporal substitution. The single-activity and IS models were adjusted for gender, age, waist circumference, and wear time. The partition model was adjusted for gender, age, and waist circumference, but not wear time.

**Table 3:** Single-activity, partition, and IS models examining associations of sedentary behavior (SB), light physical activity (LPA), and moderate-to-vigorous physical activity (MVPA) with stress.

Outcomes	Model	Target Behavior Replaced	Odds Ratio (95% CI)	Odds Ratio (95% CI)	Odds Ratio (95% CI)
			SB	LPA	MVPA
Stress (young adults 19−29)	Single-activity	-	1.00061 (1.00139−0.99982)	0.99919 (1.00042−0.99797)	0.99879 (1.00119−0.99638)
	Partition	-	1.00011 (1.00049−0.99973)	0.99921 (1.00036−0.99832)	0.99869 (1.001−0.99638)
	IS (+ 30)	SB with LPA	-	0.99912 (1.00036−0.99788)	0.99859 (1.00104−0.99615)
		SB with MVPA	-	0.99912 (1.00036−0.99788)	0.99986 (1.00104−0.99615)
		LPA with MVPA	1.00054 (1.00136−0.99973) *	-	0.99929 (1.00179−0.99678)
Stress (mid-age adults 30−49)	Single-activity	-	1.00029 (1.00071−0.99987)	0.99962 (1.00029−0.99895)	0.99943 (1.00092−0.99793)
	Partition	-	1.00013 (1.00036−0.9999)	0.99979 (1.0003−0.99929)	0.99963 (1.00109−0.99816)
	IS (+ 30)	SB with LPA	-	0.99962 (1.00029−0.99895)	0.99943 (1.00093−0.99794)
		SB with MVPA	-	0.99962 (1.00029−0.99895)	0.99943 (1.00093−0.99794)
		LPA with MVPA	1.00027 (1.00073−0.9998)	-	0.99983 (1.00147−0.99819)
Stress (older adults 50−64)	Single-activity	-	1.00000 (1.00041−0.99959	1.00012 (1.0008−0.99944)	0.99866 (1.0002−0.99712)
	Partition	-	0.99997 (1.00022−0.99972)	1.00003 (1.00048−0.99959)	0.99866 (1.00017−0.99719)
	IS (+ 30)	SB with LPA	-	1.00000 (1.00069−0.99932)	0.99866 (1.00022−0.9971)
		SB with MVPA	-	1.00000 (1.00069−0.99932)	0.99866 (1.00022−0.9971)
		LPA with MVPA	0.99991 (1.00033−0.99948)	-	0.99857 (1.00017−0.99697)

Note. * *p* < 0.05, CI = confidence interval, IS = isotemporal substitution. The single-activity and IS models were adjusted for gender, age, waist circumference, and wear time. The partition model was adjusted for gender, age, and waist circumference, but not wear time.
